# Plants of the Rubiaceae Family with Effect on Metabolic Syndrome: Constituents, Pharmacology, and Molecular Targets

**DOI:** 10.3390/plants12203583

**Published:** 2023-10-15

**Authors:** Fabiola González-Castelazo, Luis E. Soria-Jasso, Ivan Torre-Villalvazo, Raquel Cariño-Cortés, Víctor M. Muñoz-Pérez, Mario I. Ortiz, Eduardo Fernández-Martínez

**Affiliations:** 1 Laboratory of Medicinal Chemistry and Pharmacology of the Center for Research on Reproductive Biology, Department of Medicine of the School of Health Sciences, Autonomous University of Hidalgo State, Pachuca 42090, Mexico; fabiola.glz.castelazo@gmail.com (F.G.-C.); soriajasso@gmail.com (L.E.S.-J.); victor9783@hotmail.com (V.M.M.-P.); mario_i_ortiz@hotmail.com (M.I.O.); 2 Departamento de Fisiología de la Nutrición, Instituto Nacional de Ciencias Médicas y Nutrición Salvador Zubirán, Ciudad de México 14080, Mexico; ivan.torrev@incmnsz.mx

**Keywords:** diabetes, dyslipidemias, hypertension, medicinal plants, metabolic syndrome, Rubiaceae

## Abstract

Metabolic syndrome (MetS) predisposes individuals to chronic non-communicable diseases (NCDs) like type 2 diabetes (T2D), non-alcoholic fatty liver disease, atherosclerosis, and cardiovascular disorders caused by systemic inflammation, intestinal dysbiosis, and diminished antioxidant ability, leading to oxidative stress and compromised insulin sensitivity across vital organs. NCDs present a global health challenge characterized by lengthy and costly pharmacological treatments. Complementary and alternative medicine using herbal therapies has gained popularity. Approximately 350,000 plant species are considered medicinal, with 80% of the world’s population opting for traditional remedies; however, only 21,000 plants are scientifically confirmed by the WHO. The Rubiaceae family is promissory for preventing and treating MetS and associated NCDs due to its rich content of metabolites renowned for their antioxidative, anti-inflammatory, and metabolic regulatory properties. These compounds influence transcription factors and mitigate chronic low-grade inflammation, liver lipotoxicity, oxidative stress, and insulin resistance, making them a cost-effective non-pharmacological approach for MetS prevention and treatment. This review aims to collect and update data that validate the traditional uses of the Rubiaceae family for treating MetS and associated NCDs from experimental models and human subjects, highlighting the mechanisms through which their extracts and metabolites modulate glucose and lipid metabolism at the molecular, biochemical, and physiological levels.

## 1. Introduction

Non-communicable diseases (NCDs), such as type 2 diabetes (T2D), cancer, heart disease, dyslipidemia, metabolic fatty liver disease, and hypertension, were responsible for 71% (40.5 million) of worldwide deaths in 2016, making NCDs a significant public health problem. The 2030 Agenda for Sustainable Development also recognizes NCDs as a central challenge for sustainable development as they often stem from unsustainable environmental systems and practices. Therefore, various governments are committed to developing ambitious national responses to reduce premature mortality from NCDs by one-third by 2030 through prevention and treatment [1]. To achieve this goal, private and institutional health centers should have adequate diagnostic equipment, including blood pressure and glucose monitors and scales, among others, as well as essential medications such as aspirin, statins, angiotensin-converting enzyme inhibitors, diuretics, calcium channel blockers, biguanides, glucose-dependent insulinotropic polypeptide (GiP) receptor agonists, or dipeptidyl peptidase-4 (DPP4) inhibitors [2]. These medications have become the basis for the treatment of metabolic diseases. Nevertheless, it is worth noting that pharmacological treatments for NCDs can be expensive, as they often require prolonged use to achieve long-term control of blood glucose, lipids, and blood pressure [3]. As a result, it is common for health professionals and patients to explore complementary and alternative medicine options, such as acupuncture, yoga, or natural remedies based on plants with therapeutic and medicinal effects [1,2].

The use of plants for disease management has been a part of human history since ancient times. Indeed, despite advances in pharmaceutical sciences and drug development, medicinal plants continue to play a crucial role in community healthcare worldwide. It is estimated that between 350,000 and half a million species are considered medicinal plants, and approximately 80% of the world’s population opt for traditional medicine as their initial therapeutic choice [4,5]. However, it is essential to note that many plant species with medicinal properties lack scientific evidence to support their efficacy. As a result, the World Health Organization (WHO) has only listed 21,000 medicinal plants with confirmed beneficial effects [6].

Among medicinal plants, species from the Rubiaceae family are some of the most extensively studied, with hundreds of reports in the scientific literature concerning their diverse pharmacological effects and, remarkably, their therapeutical activities related to MetS, such as hypoglycemic, antihypertensive, and hypolipidemic effects. However, the latest review covering the traditional uses, phytochemistry, and biological activities of the Rubiaceae species was published in 2011, embracing just plants from the sub-Saharan region and not ones specific for treating MetS or its complications [7]. Another review published in 2016 focused on Mexican plants applied to MetS but from diverse taxonomic families [8]. Furthermore, the biological effects of the Rubiaceae family on MetS and NCDs have only been addressed from the perspective of isolated species, not from an integrative compilation of evidence; thus, a literature survey of the last twenty-five years is carried out herein to update the information on these current health topics.

Therefore, this review aims to provide an update on the species of the Rubiaceae family, focusing on the metabolic effects of constituents of these plants used for treating MetS, NCDs, and their comorbidities, collecting evidence from both experimental models and human subjects, and highlighting the biological mechanisms through which components of these plants modulate glucose and lipid metabolism at the molecular, biochemical, and physiological levels.

## 2. Metabolic Syndrome: Causes and Physiopathology

NCDs are complex and multifactorial, influenced by genetics, epigenetics, fetal programming, and environmental factors. However, a common link in the development and progression of NCDs is the presence of metabolic alterations, including obesity, insulin resistance (IR), dyslipidemia, hyperglycemia, and hypertension, which often co-occur and collectively constitute what is known as metabolic syndrome (MetS) [9]. According to the WHO, MetS is a pathological condition characterized by obesity, IR, hypertension, and hyperlipidemia [10]. The mechanisms involved in progressing from MetS to chronic diseases include IR, increased lipogenesis, altered neurohormonal signaling, oxidative stress, and chronic inflammation [8].

**Figure 1:**
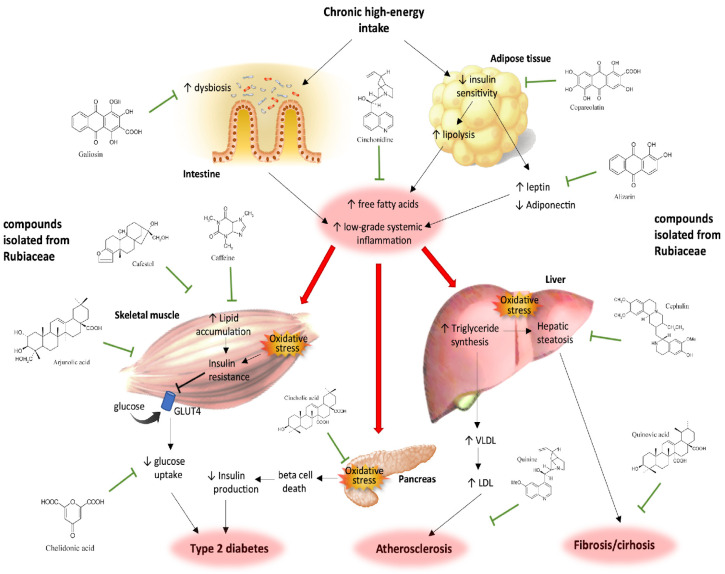
Chronic intake of high-energy diets induces intestinal microbial dysbiosis and adipose tissue hypertrophy, leading to low-grade systemic inflammation and excessive free fatty acid release into circulation. These cytotoxic molecules induce metabolic alterations in organs such as the liver, skeletal muscle, and pancreas (red arrows). Edible and medicinal plants from the Rubiaceae family contain many bioactive molecules with antioxidant, anti-inflammatory, and regulatory effects on metabolic tissues that can prevent the development and progression of non-communicable chronic diseases.

MetS is a disease caused by energy imbalance, of which a chronic high-calorie diet intake is the main causative factor. Physiologically, surplus energy is stored as triglycerides (TGs), primarily in subcutaneous adipose tissue, preventing lipid accumulation in visceral and non-adipose tissues. However, the chronic consumption of high-fat and high-carbohydrate diets and a low fruit and vegetable intake leads to dysbiosis in the intestinal microbiota and the hypertrophy of adipose tissue. An increased abundance of pathogenic bacteria augments the release of pro-inflammatory signals, such as lipopolysaccharides, leading to low-grade systemic inflammation. Adipocyte hypertrophy is characterized by unrestrained lipolysis, releasing free fatty acids into circulation, resulting in excessive fatty acid uptake and accumulation in non-adipose tissues; in turn, it induces lipotoxic tissue dysfunction, oxidative stress, and cell death. These alterations give rise to various metabolic disturbances in organs such as the liver, skeletal muscle, and pancreas, leading to metabolic diseases such as IR, hepatic steatosis, and dyslipidemia [11,12]. IR contributes to hypertension due to vasoconstriction and loss of vasodilator effects [13]. Hypertrophic visceral adipose tissue increases the secretion of the adipokine leptin, which exerts pro-inflammatory effects. The elevated circulating leptin levels associated with obesity are linked to cardiovascular risk [14]. High circulating FFA and leptin levels activate inflammatory pathways in several organs, contributing to chronic systemic low-grade inflammation, thereby increasing the risk of atherosclerosis and cardiovascular disease [15] (Figure 1).

Given the numerous altered metabolic pathways in MetS, addressing these metabolic alterations involves a range of drugs with diverse mechanisms of action. Current pharmacotherapy options encompass ATP-sensitive potassium channel inhibitors, pancreatic lipase inhibitors, hydroxymethyl-glutaryl-coenzyme A reductase (HMG CoA) inhibitors, AMP-activated protein kinase (AMPK) activators, peroxisome proliferator-activated receptors (PPAR) activators, and hypothalamic orexigenic signaling modulators. These drugs are often combined and represent the most common pharmacological approach to mitigating the progression of MetS and reducing the risk of chronic metabolic complications. However, every pharmacotherapy has known long-term adverse effects and complications and potential unknown complications. On the other hand, abundant studies have demonstrated that plants used in traditional medicine can exert beneficial metabolic effects that may prove valuable in treating the chronic diseases associated with MetS [8].

## 3. Plants Used for Treatment of Metabolic Syndrome

The preventive and therapeutic actions of medicinal plants against MetS and its associated chronic diseases can be attributed to the presence of phenolic compounds with antioxidant, anti-inflammatory, antidyslipidemic, and antihyperglycemic effects. Therefore, significant attention has been devoted to exploring the metabolic effects of medicinal plants and their bioactive substances. These compounds promise to develop new pharmaceutical products with enhanced efficacy, reduced side effects, and lower costs [8]. Various preclinical and clinical investigations have confirmed the efficacy and safety of plant species known for their antipyretic, cardioprotective, anti-inflammatory, antioxidant, antispasmodic, and immunomodulatory activities [3,4]. Good examples of medicinal plants rich in bioactive compounds are species of the Rubiaceae family, which have demonstrated promising effects in preclinical studies and clinical trials.

### 3.1. Rubiaceae Family

The Rubiaceae family ranks among the largest Magnoliopsida classes, comprising 1317 genera and 33,971 species worldwide [16,17]. In preclinical and clinical studies, plants from the Rubiaceae family have exhibited antihypertensive, lipid-lowering, antidiabetic, antioxidant, anti-inflammatory, and anticancer properties. The leaves, stems, and roots are these plants’ most commonly used parts for medicinal purposes, often prepared as powders, infusions, or decoctions [7].

The Rubiaceae family’s edible and medicinal plants contain many bioactive molecules that modulate various metabolic pathways in different organs. These metabolites exhibit structural diversity, encompassing indole alkaloids, iridoids, terpenoids, and anthraquinones, making them valuable nutraceuticals due to their beneficial effects on human health [7]. Notable among these nutraceutical polyphenols found abundantly in Rubiaceae plants are galiosin, cafestol, caffeine, arjunolic acid, chelidonic acid, cincholic acid, quinine, cephalin, quinovic acid, alizarin, copareolatin, and cinchonide. The evidence suggests that secondary metabolites derived from Rubiaceae species exert antioxidant, anti-inflammatory, and regulatory effects on metabolic tissues, attributed to their prebiotic activity, radical scavenging capacity, and immunomodulatory properties. It is worth noting that some plant polyphenols also bind and modulate the activity of metabolic transcription factors, an interaction named nutrigenomics. Nutrient-mediated regulation of gene expression adds a crucial layer of understanding regarding the molecular mechanisms through which bioactive plant molecules impact human physiology and physiopathology [18] (Figure 1).

### 3.2. Plants from the Rubiaceae Family for Treating MetS: Coffea *spp.*

Coffee is one of the most consumed beverages globally; it is widely known for its phytopharmacological properties and has been the subject of extensive research. Three species of the Coffea genus, *C. arabica*, *C. canephora*, and *C. liberica*, are used for coffee production. Whole coffee beans or their chemical constituents have shown protective effects in preclinical studies in animal models of MetS and clinical studies in subjects with MetS. For instance, a study identified nine abundant polyphenols, including 5-caffeoylquinic acid (chlorogenic acid), 3,5-di-caffeoylquinic acid, and 5-feruloylquinic acid in an aqueous extract of ground roasted coffee beans; this combination was found to reduce postprandial glucose, insulin, glucose-dependent insulinotropic polypeptide (GIP), and TGs in mice given a mixed lipid–carbohydrate emulsion. Furthermore, it was observed that this polyphenol combination modulates intestinal carbohydrate absorption by inhibiting digestive enzymes such as maltase and sucrase [19].

Interestingly, the caffeine content in the extract was not found to exert biological actions. In this regard, decaffeinated coffee had similar beneficial effects on lipid profiles and prevented platelet aggregation and blood clotting in hyperlipidemic rats to that containing caffeine [20]. Moreover, the metabolic effects of ethanolic extracts from unroasted *Coffea canephora* robusta and *Coffea arabica* beans were assessed both in vivo using high-fat diet (HFD)-fed mice and in vitro using an insulin-induced adipogenesis model in 3T3-L1 preadipocytes. Mice fed an HFD who received the Robusta bean extract showed a reduced body weight and fat content, improved liver steatosis, and better profiles of circulating lipids, glucose, and leptin; additionally, the epididymal adipocytes were smaller, indicating a preventive effect on adipocyte hypertrophy. Indeed, two concentrations of the ethanolic extract lowered adipogenesis in cultured adipocytes by down-regulating the adipogenesis-related genes adiponectin and PPAR-γ [21]. However, it is remarkable that the reduction in PPAR-γ and adiponectin observed in vitro may not directly translate to in vivo effects, as this could lead to adipocyte dysfunction and MetS. Therefore, the in vitro effects of a particular molecule must be carefully interpreted by assessing their systemic effects through in vivo studies. In another study, the acute and chronic effects of a mixture of caffeic acid, trigonelline, and cafestol were examined in a MetS model involving rats fed an HFD or a high-fructose diet. Chronic administration of this mixture did not significantly affect body weight or glycemia but did improve postprandial hyperinsulinemia, IR, and plasma adiponectin levels. The activity of the liver damage marker alanine aminotransferase (ALT) was diminished in plasma and the liver. The hepatic content of TGs was also lower. Conversely, acute administration of the mixture increased postprandial glycemia. The findings suggested that long-term consumption of this coffee nutraceutical mixture enhances insulin sensitivity and provides hepatoprotective effects in rats with MetS [22].

The aqueous extract of *Coffea arabica* pulp is rich in chlorogenic acid, caffeine, and epicatechin, and has been found to have cholesterol-lowering properties. This extract has been shown to inhibit the transport of cholesterol micelles in both Caco-2 cells (human epithelial colorectal adenocarcinoma cells) and rat jejunal loops in a concentration-dependent but non-competitive way. Moreover, the administration of this extract has been shown to prevent excessive weight gain and improve the plasma lipid profile in hypercholesterolemic rats fed an HFD. The proposed mechanism of action for the pulp extract and its primary constituents involves the activation of the nuclear receptor liver X receptor-α (LXRα). In turn, the activation of this nuclear receptor down-regulates the gene expression of the cholesterol transporter Niemann–Pick C1 (NPC1) in intestinal cells and increases the expression of CYP7A1 in the liver. These actions increase hepatic bile acid synthesis and intestinal cholesterol excretion [23].

Growing evidence suggests that the metabolic alterations associated with MetS have an inflammatory origin. Activating inflammatory responses in adipose tissue, liver, and vasculature leads to adipose tissue dysfunction, steatohepatitis, and atherosclerosis [23]. Recent research has identified colonic microbiota as a source of inflammatory signals, such as lipopolysaccharides and trimethylamine. Pathogenic bacteria release those molecules into the colonic lumen, and their abundance is closely linked to unhealthy dietary habits. A reduced intake of prebiotic molecules, including resistant starches, non-digestible polysaccharides, and polyphenols, induces dysbiosis (or dysbacteriosis), characterized by shifts in intestinal bacterial abundance and a reduction in taxa diversity. Intestinal dysbiosis is critical in altering various metabolic pathways in different organs. Therefore, restoring a healthy diversity of colonic microorganisms is essential in treating MetS [24].

Recent studies have evaluated the effect of coffee intake on microbiota diversity and its impact on MetS. In one study, an aqueous green coffee extract was tested in apolipoprotein E-deficient (ApoE-/-) mice fed an atherogenic diet, a well-established experimental model for studying cardiovascular and metabolic diseases. While the extract did not reduce the progression of atherosclerotic lesions or plasma lipid levels in the ApoE-/- mice, it effectively corrected key metabolic parameters, such as fasting glucose levels, IR, serum leptin levels, urinary catecholamines, and liver TGs. Additionally, the administration of the extract led to reduced weight gain, reduced adiposity, lowered inflammatory infiltrate in adipose tissue, and prevented liver damage [23,24].

Furthermore, the extract increased hepatic interleukin (IL)-6 and total serum IgM, which correlated with alterations in intestinal microbiota composition [24]. In another study, spent coffee grounds containing chlorogenic acid, caffeine, trigonelline, and diterpenes were supplemented to rats fed either an HFD or a high-carbohydrate diet (HCD) to induce MetS. Including spent coffee grounds reduced body weight, diminished abdominal and total body fat accumulation, lowered systolic blood pressure, and decreased plasma TGs and free fatty acids. Moreover, spent coffee grounds also improved glucose tolerance and cardiac and hepatic function. These positive changes were associated with increased diversity in the gut microbiota and a reduction in the Firmicutes to Bacteroidetes phyla ratio, linked to improvements in MetS-related alterations [25].

The therapeutic effects of coffee on MetS have also been evaluated in clinical trials. Regular consumption of a green/roasted coffee blend has been found to positively affect blood pressure, glucose, and triglyceride levels in healthy individuals and hypercholesterolemic subjects, thereby preventing the development of MetS. These beneficial metabolic effects are attributed to reduced levels of pro-inflammatory factors such as leptin, plasminogen activator inhibitor-1 (PAI-1), and resistin. These effects are, in part, mediated by the activity of hydroxycinnamic acids (caffeoylquinic acids, mostly 5-O-caffeoylquinic acid) and methylxanthines (such as caffeine) present in coffee beverages [26]. Other clinical approaches have shown that patients with MetS and administered with capsules containing a 400 mg hydro-alcoholic extract of decaffeinated green coffee beans experienced improvements in several MetS components, such as high systolic blood pressure, elevated fasting blood glucose, IR, and abdominal obesity, which are the main etiological factors of MetS [25]. The capsules contained a variety of compounds, including 5-caffeoylquinic acid (35–40%), 3-caffeoylquinic (10–15%) acid, 4-caffeoylquinic acid, 3,4-di-caffeoylquinic acid, 3,5-di-caffeoylquinic acid, 4,5-di-caffeoylquinic acid, 3-feruloylquinic acid, 4-feruloylquinic acid, and 5-feruloylquinic acid. These capsules were administered twice a day for eight weeks [27]. The report also suggests that the capsules can help reduce appetite, preventing excessive food intake [27].

In another study, an extract of medicinal plants, including decaffeinated *Coffea canephora* beans (containing 30 mg of chlorogenic acid), was given to patients with non-alcoholic fatty liver disease (NAFLD) associated with IR and diabetes. This extract effectively reduced serum glucose and insulin levels, improved IR, and alleviated hepatic steatosis. These improvements were correlated with an increased expression of insulin receptors in the liver [28]. A comprehensive review of coffee’s effects on MetS and its components is recommended [29].

While coffee offers potential benefits for MetS, it is vital to consider its potential risks due to its high caffeine content, including insomnia, tremulousness, palpitations, anxiety, and headaches. In women, coffee may also increase the risk of fractures, and during pregnancy, coffee consumption is associated with an elevated risk of low birth weight and preterm labor. Therefore, limiting coffee consumption to three to four cups daily is advisable, as this appears to be a safe range associated with the most robust beneficial effects [30] (Table 1).

### 3.3. Plants from the Rubiaceae Family for Treating MetS: Exosterma *spp.*

The genus Exosterma comprises twenty-five species of trees and shrubs. *Exosterma caribaeum* and *E. mexicanum* are traditionally used for treating malaria, fever, and high blood glucose [31,32]. These plants are rich in coumarins and phenylcoumarins, with identified compounds such as 5-O-B-D-galactosyl-7-methoxy-3’,4’-dihydroxy-4-phenylcoumarin; 7,4’,5’-trihydroxy-4-pheny1-5,2’-oxidocoumarin and 7,4’-dimethoxy-5’-hydroxy-4-phenyl-5,2’-oxido-coumarin; and monoterpenoid indole alkaloids such as phenyl styrene, chloroquine, quinine, and chlorogenic acid [33,34]. Notably, chlorogenic acid has been found to exert hypocholesterolemic and antihyperglycemic effects [35]. Coumarins isolated in plants of this genus include 8-hydroxy-5,7,40-trimethoxy-4-phenylcoumarin (exostemin I) and 5,7,8,40-tetramethoxy-4-phenylcoumarin (exostemin III), both of which have been shown to inhibit the activity of the 3,5’-cyclic adenosine monophosphate (cAMP) phosphodiesterase [36]. This effect is clinically relevant since cAMP is an intracellular second messenger in various metabolic pathways and immune cell activities. The cAMP signaling pathways can exert pro- or anti-inflammatory effects depending on cell type [37]. Indeed, plants of the Exosterma genus have traditionally been used in various preparations to treat T2D, a disease with a prevalence of 17% in Mexico. Some extracts from *E. caribaeum* have demonstrated antihyperglycemic effects in rodent models of diabetes. For instance, a CH_2_Cl_2_/MeOH extract showed a significant reduction in blood glucose levels, around 17%, with 100 and 300 mg/kg doses [33]. Aqueous extracts also induced significant antihyperglycemic effects in nicotinamide/streptozotocin (NA/STZ)-induced diabetic mice when compared to the control group [36] (Table 1).

### 3.4. Plants from the Rubiaceae Family for Treating MetS: Hamelia *spp.*

*Hamelia patens* is among the most extensively studied genera within the Rubiaceae family due to its reported antihyperglycemic effect [38,39,40]. Various extracts derived from distinct parts of the plant have shown a wide range of activities, including antimicrobial [41,42,43], anti-inflammatory [39,41], antioxidant [44], antidepressant [45], antinociceptive [46], hepatoprotective [47], and cytotoxic activities [48,49]. Several metabolites have been isolated from *H. patens*, including flavonoids, terpenoids, coumarins, sterols, and anthocyanins [50]. These molecules exert significant antioxidant activities, preventing oxidative-stress-induced cellular damage [51]. In one study, an alcoholic extract of *H. patens* phytochemicals was evaluated in HepG2 cells to assess its antioxidant and hepatoprotective capacity. The cells were subjected to oxidative damage induced by the cytotoxic agent CCl_4_. The *H. patens* extract effectively countered oxidative stress and prevented cell lysis, demonstrating a direct association between the radical-scavenging capabilities of the extracts and their hepatoprotective effects [47]. These findings are particularly relevant as the pathogenesis of T2D is associated with the generation of reactive oxygen species (ROS), which favors the generation of toxic metabolic by-products such as advanced glycation end-products and lipid peroxides, ultimately resulting in IR in skeletal muscle and liver. Moreover, in a pro-inflammatory environment, oxidative stress can contribute to hepatocyte necrosis, leading to hepatic steatohepatitis and cirrhosis. Hence, using antioxidant phytochemicals like those found in *H. patens* may offer a non-pharmacological therapeutic approach to preventing liver damage and T2D [52].

Another study demonstrated that the administration of ethanolic or petroleum ether extracts of *H. patens* led to reduced blood glucose levels, total cholesterol, and circulating TGs in a rodent model of alloxan-induced diabetes [53]. In managing T2D, α-glucosidase inhibitors are commonly prescribed to delay the digestion and absorption of carbohydrates, thus reducing postprandial hyperglycemia [54]. One study investigated how *H. patens* hexane extract inhibits the α-glucosidase activity in vitro [45]. These effects were attributed to the presence of compounds like β-sitosterol and stigmasterol [38], as well as other identified compounds in *H. patens*, including acarbose, quercetin, epicatechin, kaempferol, and naringenin, which are known for their effectiveness in inhibiting the enzyme α-glucosidase [55]. Acarbose, a well-established α-glucosidase inhibitor, has been successfully employed to manage hyperglycemia with satisfactory results since it carries a minimal risk of hypoglycemia. Interestingly, this compound has also demonstrated the ability to reduce circulating TGs, thereby mitigating cardiovascular risk [56,57] (Table 1).

### 3.5. Plants from the Rubiaceae Family for Treating MetS: Ixora *spp.*

*Ixora coccinea* L. is a flowering plant known for its various medicinal properties, including analgesic and wound-healing effects, as well as antioxidant, antibacterial, anti-inflammatory, antidiarrheal, hepatoprotective, cardioprotective, antimutagenic, antitumor, and antihypertensive activities. This genus contains a diverse range of compounds, including peptides (ixorapeptides), triterpenoids, alkaloids, resins, saponins, steroids, fatty acids, and the polyphenols proanthocyanidins, flavonoids, flavonoids, glycosides, and tannins. Some primary compounds in *I. coccinea* are lupeol, oleic acid, linolic acid, ursolic acid, oleanolic acid, stearic acid, and sitosterol. The flowers of this plant contain rutin, leucocyanidin glycoside, cyanidin-3-rutinoside, and delphinidin monoglycoside. The root bark contains octadecadienoic acid, while the root oil contains palmitic, oleic, and stearic acids and linoleic acid methyl esters. The leaves are rich in ixoratannin A-2, epicatechin, procyanidin A2, cinnamotannin B-1, and various flavonoids and glycosides, such as kaempferol-7-o-α-1-rhamnoside, kaempferol-3-o-α-1-rhamnoside, quercetin-3-o-α-1-rhamnopyranoside, kaempferol-3,7-o-α-1-dirhamnoside, and quercitrin. The leaves of *I. coccinea* and those of *I. parviflora* have demonstrated potent lipid-lowering and antioxidant activities that may contribute to their cardioprotective and antiatherosclerotic effects [58,59]. Indeed, the aqueous extract of *I. coccinea* leaves has been shown to possess antihyperglycemic and hypolipidemic properties in rats with alloxan-induced diabetes. Furthermore, the methanolic extract of its flowers inhibited both α-amylase and α-glucosidase enzymes, indicating its potential to prevent postprandial hyperglycemia in individuals with T2D [60,61] (Table 1).

### 3.6. Plants from the Rubiaceae Family for Treating MetS: Hintonia latiflora

Copalchi, also known as “palo amargo” (sour stick) in Latin America, is derived from the bark of the Hintonia latiflora tree (syn. Coutarea latiflora). It has primarily been utilized as an alternative therapy for individuals with T2D due to its demonstrated hypoglycemic properties. The antihyperglycemic effect of *H. latiflora* bark can be attributed to its secondary metabolites, such as 4-phenyl coumarins, glycosides, cucurbitacins, glycosides, and coutareagenin, which have been reported as its main bioactive compounds [18,33,62,63]. Iridoids are regarded as one of the most crucial bioactive compounds found in copalchi due to their significant antioxidant and anti-inflammatory effects, which contribute to its cardioprotective, antihyperglycemic, hypolipidemic, and hepatoprotective effects [64]. Iridoid-rich extracts of copalchi have been evaluated in rodents, showing antihyperglycemic effects, demonstrating their potential as a non-pharmacological treatment for individuals with T2D (Figure 1) [33,65]. Recent studies have unveiled that, beyond their anti-inflammatory and antioxidant activities, iridoids also modulate the expression of transcription factors involved in lipid metabolism regulation, such as peroxisome proliferator-activated receptors (PPARs) and sterol regulatory element-binding proteins (SREBPs) [23,24] (Table 1).

### 3.7. Plants from the Rubiaceae Family for Treating MetS: Miscellaneous Members

The Alibertia genus includes diverse medicinal plants; for example, Alibertia edulis is used for treating snake bites; however, its aqueous extract has antioxidant, antihypertensive, diuretic, and hypoglycemic effects as observed in high-fat-diet (HFD)-fed mice [66,67]. Also, A. edulis is not toxic even at high doses despite its diverse metabolite content, which includes iridoids, saponins, oleanane diterpene, alkaloids, and tannins [68] (Table 1).

Nauclea latifolia has many ethnomedicinal applications covering many diseases, including infections and malaria. In many experimental animal models, N. latifolia has been found to exert antihepatotoxic, antinociceptive, anti-inflammatory, antipyretic, and anti-hyperglycemic/antidiabetic effects. Ethanol extracts of N. latifolia leaves contain a variety of phytochemicals, including alkaloids, flavonoids, steroids, glycosides, and saponins [69,70]. Extracts rich in alkaloids from N. latifolia leaves have exhibited beneficial metabolic effects such as antidiabetic/antihyperglycemic, hypolipidemic, and hypocholesterolemic properties (Table 1).

*Morinda citrifolia*, commonly known as “noni,” is a rich source of phytochemicals, containing approximately two hundred phenolic compounds, including anthraquinones, flavonoids, iridoids, ketones, lignans, carbohydrates, organic acids, alcohols, vitamins, triterpenoids, nucleosides, sterols, fatty acids, carotenoids, among others [71]. These bioactive secondary metabolites are found in various parts of the noni plant, including the fruit. Consumption of *M. citrifolia* fruits, often as pasteurized juice, either alone or mixed with other juices, effectively prevents metabolic dysfunctions. Its metabolic activities include regulating body weight and fat content, improving lipid and glucose metabolism, and lowering elevated blood pressure. Noni fruit has also been shown to exert prebiotic and hepatoprotective effects. *M. citrifolia* leaves have been associated with decreased abdominal fat and TGs, increased adiponectin levels, and changes in adipose tissue cell dynamics [72]. Furthermore, an oral administration of aqueous extract from dried *M. citrifolia* to Wistar rats with sucrose-induced MetS resulted in an antiobesity and antidyslipidemic effect associated with increased adiponectin levels [73,74] (Table 1).

Within the Uncaria genus, the *U. tomentosa* species has a traditional history of preventing and treating different metabolic alterations. An extract derived from *U. tomentosa* has demonstrated the ability to enhance insulin sensitivity while reducing liver inflammation and hepatic fat accumulation in HFD-fed and ob/ob mice. These findings suggest that *U. tomentosa* could be considered as a potential therapeutic intervention for individuals with obesity to enhance hepatic insulin sensitivity and reverse non-alcoholic steatohepatitis (NASH) to a less harmful NAFLD [75]. In an in vivo study, several rat models evaluated the pharmacological effects and toxicity of a commercial hydro-alcoholic extract, GlucoMedix^®^, derived from *Stevia rebaudiana* (Asteraceae) and the pentacyclic chemotype of *U. tomentosa* (Willd.), proving to be safe and effective for use as a treatment for MetS [76]. In a prospective multicenter clinical trial, the traditional Chinese herbal formula known as Jiangzhuoqinggan (JZQG), composed of rhubarb, Coptis, Cassia, and Uncaria, demonstrated therapeutic effects in patients with both hypertension and MetS [77] (Table 1).

The aqueous extract of Alseis yucatanensis bark also has antihypertensive activity, as assessed in an isolated rat aorta, where the authors suggested that the extract blocks calcium channels, resulting in a sustained vasorelaxant effect by opening K^+^ channels [78]. There are reports regarding the antihyperglycemic activity of *Bouvardia ternifolia*, which contains two triterpenes: ursolic and oleanolic acids. These compounds, isolated from the chloroform extract of the dried stem, have been shown to lower blood glucose levels in alloxan-induced diabetic mice [79,80] (Table 1).

Geniposide, which is an iridoid glycoside from the fruit of *Gardenia jasminoides*, was tried in spontaneously obese type 2 diabetic mice, showing a suppression of body weight and visceral fat accumulation, alleviation of abnormal lipid metabolism, and suppression of intrahepatic lipid accumulation [81].

**Table 1:** Pharmacological effects of genera and species from Rubiaceae family on MetS.

Genus	Species	Extract, Dosage Form, and Doses	Chemical Constituents (Secondary Metabolites)	Experimental Model or Clinical Disease	Effects and Mechanism of Action Suggested	References
Alibertia	edulis	Lyophilized aqueous extract of leaves (a) 20–200 mg/kg for 1 and 7 days, p.o. (b) 200–400 mg/kg for 2 weeks, p.o.	Caffeic acid, quercetin 3-rhamnosyl-(1→6)-galactoside, iridoid ixoside, ferulic acid, and rutin	(a) Renovascular hypertension by two-kidney, one-clip Goldblatt model (b) High-fat-diet (HFD)-fed mice	(a) Antihypertensive, diuretic by high excretion of Na^+^, K^+^, Cl^-^, and Ca^++^. Antioxidant effects by free radical scavengers (b) Anti-hyperglycemic effect by regulation of IKKb/NF-κB pathway as a mediator of IR and insulin sensitization	[66,67,68]
Alseis	yucatanensis	Aqueous extract of bark. Blocking of calcium channels, internal ED50 = 0.49 mg/mL, and external ED50 = 2.34 mg/mL- Relaxation of norepinephrine-contracted ED50 = 0.12 and KCl-contracted EC50 = 1.73 mg/mL	Not reported	Relaxation of isolated endothelium-denuded rat aortic tissues	Anti-hypertensive, by blocking of Ca^++^ channels with a sustained vasorelaxant effect by opening K^+^ channels	[74]
Bouvardia	ternifolia	(a) Chloroform extract of dried stem at 100, 200, and 300 mg/kg, i.p. (b) Pure compounds at 25, 50, and 75 mg/kg, i.p.	Two triterpenes, ursolic and oleanolic acids	(a) and (b) Alloxan-induced diabetic mice	(a) and (b) Extract and pure compounds lowered blood glucose levels in normal and diabetic mice	[78,79]
Coffea	arabica	(a) Aqueous extract of ground roasted coffee beans, 200 mg/kg, intragastric tube (b) Pure compound, caffeic acid 71.4 mg/kg, trigonelline 47.6 mg/kg, and cafestol 2.4 mg/kg, p.o., a day per rat for 12 weeks (c) Aqueous extract of pulp, 5–200 mg/mL CPE, with chlorogenic acid 2.33 mg/mL and caffeine 0.79 mg/mL in vitro. For in vivo, 1000 mg/kg a day for 12 weeks (d) Five % of spent coffee grounds in diet for 8 weeks (e) Soluble green/roasted coffee product mixture (35:65, *w/w*), 2 g serving of coffee blend three times a day for 8 weeks (f) Capsules with 400 mg of green coffee hydro-alcoholic extract containing 2400 mg of green coffee bean and 186 mg of chlorogenic acid twice daily for 64 days. 53.8% of total polyphenols per capsule	(a) 5-caffeoylquinic acid (chlorogenic acid), 3,5-dicaffeoylquinic acid, and 5-feruloylquinic acid (b) Caffeic acid, trigonelline, and cafestol (c) Chlorogenic acid, caffeine, and epicatechin (d) Chlorogenic acid, caffeine, trigonelline, and diterpenes (e) Hydroxycinnamic acids (caffeoylquinic acids, mostly 5-O-caffeoylquinic acid) and methylxanthines (caffeine) (f) Caffeoylquinic acid (35–40%), 3-caffeoylquinic acid (10–15%), 4-caffeoylquinic acid, 3,4-dicaffeoylquinic acid, 3,5-dicaffeoylquinic acid, 4,5-dicaffeoylquinic acid, 3-feruloylquinic acid, 4-feruloylquinic acid, and 5-feruloylquinic acid	(a) Mice administered with a mixed lipid–carbohydrate emulsion, 4 g/kg, intragastric tube (b) MetS in rats caused by HFD and high-fructose diet (c) Cholesterol micelle transport in cultivated Caco-2 cells and rat jejunal loops, and hypercholesterolemic HFD-fed rats (d) MetS in HFD- and H-carbohydrates-D-fed rats (e) Healthy and hypercholesterolemic subjects to prevent MetS (f) Patients diagnosed with MetS	(a) Reduced postprandial glucose, insulin, glucose-dependent insulinotropic polypeptide (GIP), and TGs. Inhibition of digestive enzymes such as maltase and sucrase modulates complete lipid and carbohydrate oxidation (b) Improved fed hyperinsulinemia, IR, and plasma adiponectin levels. Diminished plasma and liver ALT, besides hepatic TG and steatosis. Pronounced postprandial glycemia after acute administration. Effects of enhanced insulin sensitivity and hepatoprotection (c) Inhibition of cholesterol transport in the cells and jejunal loops in a concentration-dependent but non-competitive way. Diminishing of body weight and weight gain, improved plasma lipid profiles by activation of LXRα and down-regulation of NPC1L1 (d) Reduction of body weight, abdominal and total fat, systolic blood pressure, plasma TG and non-esterified fatty acids. Enhanced glucose tolerance and heart and hepatic function. Changes correlated with an increased diversity of the gut microbiota and the reduced ratio of Firmicutes to Bacteroidetes phyla (e) Lowered blood pressure, glucose, and triglyceride levels may be due to reduced leptin, plasminogen activator inhibitor-1 (PAI-1), and resistin levels (f) Improved MetS components, high systolic blood pressure, fasting blood glucose, and MetS etiological factors comprising IR and abdominal obesity. Capsules could reduce appetite level in patients	[19,22,23,25,27]
	canephora robusta	(a) Ethanolic extracts of unroasted beans, 20–500 µg/mL for cell culture and 330 mg/kg a day for 25 days for the in vivo model (b) Aqueous extract of green coffee beans, equivalent to 220 mg/kg of chlorogenic acid trice a week for 14 weeks, intragastric (c) Extract of a mixture of medicinal plants with 30 mg of chlorogenic acid from *C. canephora* in 1 tablet per day for six months	(a) Caffeine, caffeic acid, and caffeic quinic acids (b) Caffeine, caffeic acid, chlorogenic acid, and their quinic derivatives (c) Chlorogenic acid among diverse from other species	(a) In vitro insulin-induced adipogenesis in 3T3-L1 preadipocytes and in vivo HFD-fed mice (b) Atherogenic diet-fed apolipoprotein E-deficient (ApoE-/-) mice (c) Patients with NAFLD associated with IR and diabetes	(a) Reduced blood concentrations of lipids, glucose, leptin, body and fat weight, improved liver steatosis, and smaller epididymal adipocytes. Lowered adipogenesis in adipocytes by down-regulation of adipogenesis-related genes adiponectin and PPAR-γ (b) Improved fasting glucose, IR, serum leptin, urinary catecholamines, and liver TGs, diminished weight gain, adiposity, inflammatory infiltrate in adipose tissue, and hepatoprotection, all by immune-stimulatory effect increasing hepatic IL-6 and total serum IgM, which induced shifts in intestinal microbiota (c) Reduction in serum glucose, insulin levels, IR, and hepatic steatosis correlated with an increased serum insulin receptor expression	[21,24,28]
Exostema	caribaeum	Macerated CH_2_Cl_2_–MeOH (1:1) of stem bark (a) 10, 30, 100, 300 mg/kg for 30 days, p.o. (b) 1. Aqueous extract of *Exostema caribaeum* stem bark (500 mg/kg). 2. In oral sucrose tolerance test, the same aqueous extract (100, 300 and 500 mg/kg)	(a) Cucurbitacins and 4-phenylcoumarins, phenyl styrene, as well as an indole monoterpenoid alkaloid (b) Chlorogenic acid, seven 4-phenylcoumarins	(a) STZ-induced T2D in rats by a single i.p. injection (b) Nicotinamide/streptozotocin (NA/STZ)-diabetic mice	(a) Antihyperglycemic effect could be due to insulin release stimulation that might increase renewal of β-cells or permit recovery of partially destroyed β-cells stimulating pancreatic insulin secretion (b) 1. Causes a significant antihyperglycemic effect similar to glibenclamide 2. Significant decrease in the postprandial glycemia peak	[33,36] [34,36]
Hamelia	patens	(a) Hexane, DCM–EtOAc, MeOH–EtOAc, and MeOH–aqueous (b) MeOH crude extract and fractional MeOH extracts; 35, 75, and 150 mg/kg (c) Water (30 and 300 mg/kg) and ethanol-water (60 and 600 mg/kg) extracts (d) Ethanolic extract 100 mg/kg and 400 mg/kg petroleum ether extract 100 mg/kg and 400 mg/kg. Extracts were administered orally for 20 days	(a) β-sitosterol and stigmasterol (b) Acarbose, ursolic acid, quercetin, epicatechin and chlorogenic acid (c) Chlorogenic acid, caffeic acid, and quercetin. Rutin was not detected (d) Not reported	(a) α-glucosidase inhibition in vitro (b) Wistar rats induced with streptozotocin and in vitro α-glucosidase inhibitory activity (c) NA/STZ-induced hyperglycemic Wistar rats (d) Alloxan-induced diabetic Wistar rats	(a) Significant decrease in blood glucose level and total cholesterol triglyceride level in model of alloxan-induced diabetes (b) Extract hexane showed α-glucosidase inhibition (c) Both extracts exert antihyperglycemic effect at doses tested. Water extract produces statistically significant effect at 120 min, while ethanol-water extract produces antihyperglycemic effect at 60 min (d) Extracts at 150 mg/kg dose showed more significant decrease in glucose level and serum insulin compared to diabetic control. High α-glucosidase inhibitory activity was found in both extracts	[38] [27] [40] [42]
Ixora	coccinea	(a) Aqueous extract of leaves administered orally with doses of 100, 200, and 400 mg/kg (b) MeOH extract of flowers (20–100 mg/mL)	(a) Alkaloids, tannins, saponins, flavonoids, anthraquinones, anthocyanosides, and reducing sugars in the extract (b) Flavonoids, phenols, steroids, and tannins	(a) Wistar rats with alloxan-induced diabetes (b) In vitro inhibition of α-amylase and α-glucosidase enzymes	(a) Antihyperglycemic and hypolipidemic (lowered TGs and cholesterol) effects of aqueous extract (b) MeOH extract of flowers inhibited up to 70–72% of both the α-amylase and α-glucosidase enzymes in a concentration-dependent manner, suggesting an in vitro antidiabetic effect	[58,59,60,61]
Nauclea	latifolia	Ethanol extract of leaves	Flavonoids, phenols, saponins, and sterols	Sixty overnight-fasted rats were injected intraperitoneally with 150 mg/kg alloxan for diabetes induction	Decreased total cholesterol, LDL cholesterol, and fasting blood glucose concentrations with improved level of HDL by fractions indicates that fractions have antihyperglycemic and hypolipidemic activity	[70]
Morinda	citrifolia	Noni fruit and other parts of the plant Noni fruit juice Ethanolic extract of leaves Aqueous fruit extract	Phenolic compounds, anthraquinones, flavonoids, iridoids, ketones, lignans, triterpenoids, nucleosides, sterols, fatty acids, carbohydrates, organic acids, alcohols, vitamins, and carotenoids; iridoids, flavonoid (rutin), 18 novel trisaccharide fatty acid esters	Mice received 1.5 µL/g twice daily/5 weeks Rats 250 mg/mL/9 weeks 500 mg/mL/9 weeks 250 and 500 mg/kg of *Morinda citrifolia* in high-fat/high-fructose-fed Swiss mice	It reduced body weight by 40% in mice fed control and 25% in HFD mice. It reduced adipose tissue weights and plasma TG and improved glucose tolerance. Positive effects on adiposity, fecal fat content, plasm lipids, insulin, and leptin levels, especially MLE 60 and 500 mg/kg. Both concentrations of extract improved the metabolic perturbations caused by obesity. AE at 500 mg/kg downregulated hepatic PPAR-γ, SREBP-1c, and fetuin-A mRNA but upregulated PPAR-α mRNA in white adipose tissue; hypoglycemic effects could be associated with de novo expression of genes involved in lipogenesis	[72,73,74]
Uncaria	tomentosa	Crude plant extract: 50 mg/kg/5 days in both obese mouse models: C57BL/6 HFD and genetically obese (ob/ob+). GlucoMedix^®^ (Uncaria and Stevia): Daily oral doses of 250–1000 mg/kg	Uncarine D, uncarine F, mitraphylline, isomitraphylline, uncarine C, and uncarine E Stevia (e.g., steviol glycosides) and Uncaria (e.g., pentacyclic oxindole alkaloids, lacking of tetracyclic oxindole alkaloids)	(a) Genetically obese mice (ob/ob+) High-fat-diet (HFD) fed C57BL/6 mice Effects in hyperglycemic (alloxan)/28 days, hyperlipidemia (cholesterol) 21 days, and hypertensive (L-NAME)/28 days in rat models.	Significantly reduced liver steatosis and inflammation in both models: (a) Liver of ob/ob+ group had reduced levels of phosphorylated Ikkβ and NFκB compared to ob/ob+ vehicle mice, at 80% and 20%, respectively, *p* < 0.05. But it did not affect TNF-α expression (b) Treatment induced a substantial reduction in TNF-α, levels of F4/80 mRNA, and reverted enhanced IL-1β and induced enhancement of IL-10 and arginase-1 expression. Subacute oral toxicity was >2000 mg/kg. GlucoMedix^®^ was a safe and effective treatment for hyperglycemia, hyperlipidemia, and hypertension	[75] [76]
Chinese herbal medicine formula Jiangzhuoqinggan (JZQG)	Formula consists of rhubarb, Coptis, Cassia, and Uncaria	For the JZQG treatment group, 170 mL of heated decoction was administered twice a day orally. For the irbesartan control group, a 150 mg tablet (Sanofi-Aventis Hangzhou Minsheng Pharmaceutical Co., Ltd., Hangzhou, China) was given orally once per day for 4 weeks	Berberine, palmatine, cory noxeine or isocory noxeine, coptisine, magnoflorine, epiberberine, and rhynchophylline compounds can also be used as quality control markers for JZQG decoction	240 subjects, ages 18–65 years old, having mild to moderate hypertension with the following characteristics: (1) TG 150 mg/dL or have received antidyslipidemia treatment (2) HDL-C): men < 40 mg/dL, women < 50 mg/dL, or have received the related treatment (3) Fasting plasma glucose (FPG) 100 mg/dL, diagnosed type 2 diabetes, or have received glycemic control treatment, and (4) TCM diagnosed for liver and stomach damp heat syndrome. Patients were distributed into JZQG group and irbesartan group	There was a significant reduction in systolic and diastolic blood pressure in JZQG group (*p* < 0.01). JZQG group showed a more significant reduction in systolic and diastolic blood pressures at 24 h than irbesartan group. A significant difference in waist circumference was observed in JZQG group but not in irbesartan group	[77]

## 4. Concluding Remarks

MetS is a prevalent health condition in many countries worldwide, and it significantly increases the risk of developing chronic degenerative diseases such as T2D, NAFLD, atherosclerosis, and cardiovascular diseases. The molecular, biochemical, and pathophysiological mechanisms involved in the development of MetS and its progression into chronic diseases encompass several factors. These include systemic inflammation induced by intestinal dysbiosis, diminished antioxidant capacity resulting in the accumulation of reactive oxygen species, oxidative stress, and reduced insulin sensitivity in the liver, skeletal muscle, and adipose tissue. These factors collectively lead to hyperglycemia and dyslipidemia. Plant-derived metabolites exert prebiotic, anti-inflammatory, antioxidant, antihyperglycemic, antihyperlipidemic, and insulin-sensitizing activities. These bioactive compounds with beneficial health effects, often called nutraceuticals, are the basis of traditional herbal medicine. Within the Rubiaceae family of plants, numerous bioactive molecules have been extensively studied for their potential to prevent chronic low-grade inflammation, oxidative stress, IR, and hepatic lipid accumulation. As a result, the beneficial metabolic effects of plants from the Rubiaceae family hold promise for developing novel non-pharmacological approaches to preventing and treating MetS and its associated chronic complications. This review highlights the potential of bioactive compounds derived from the Rubiaceae family of plants to improve public health by addressing the growing challenges that MetS and its related diseases pose.
